# A Commentary on Gender Does Not Matter: Add-on Repetitive Transcranial Magnetic Stimulation Treatment for Female Methamphetamine Dependents

**DOI:** 10.3389/fncir.2020.00027

**Published:** 2020-06-17

**Authors:** Yan Zeng, Hui Zheng, Weiqi He

**Affiliations:** ^1^Research Center of Brain and Cognitive Neuroscience, Liaoning Normal University, Dalian, China; ^2^Key Laboratory of Brain and Cognitive Neuroscience, Liaoning, China; ^3^Shanghai Key Laboratory of Psychotic Disorders, Shanghai Mental Health Center, Shanghai, China

**Keywords:** gender difference, female, mathamphetamine dependents, rTMS, cortical plasticity

Repetitive transcranial magnetic stimulation (rTMS) is a non-invasive brain neuromodulation approach, which has demonstrated clinical efficacy for major depressive disorder, obsessive-compulsive disorder, stroke, and substance use disorders (Mantovani et al., 2006; O'Reardon et al., 2007; Ludemann-Podubecka et al., 2015; Shen et al., 2016). In addition, rTMS intervention could enhance cognitive performance (e.g., inhibit control, decision making, working memory) in healthy individuals when targeting relative networks (Bagherzadeh et al., 2016; Rahnev et al., 2016; Lowe et al., 2018). One important neural mechanism underlying these efficacy was the cortical plasticity induced from the rTMS stimulation.

Previous studies uncovered potential gender differences in rTMS induced cortical plasticity. The study conducted by Inghilleri and colleagues revealed ovarian hormones dependent effect of primary motor cortex (M1) excitability is present in females, whereas this is not present in males (Inghilleri et al., 2004). The results indicated that females showed higher rTMS induced motor evoked potential (MEP) in the late menstrual cycle, which is consistent with the study by Smith and his colleagues (Smith et al., 1999, 2002). However, the difference of cortical excitability induced by TMS in male and female was not been found in young adulthood in other studies, but age-related cortical excitability discrepancy within gender shows that females progress more reduction of excitability in elderly age compared to male (Sella et al., 2014; Polimanti et al., 2016). One reason for the absence of plasticity gender difference in young adulthood was that the menstrual cycle of female subjects was not considered. TMS could alter cognitive performance; females displayed reduced smile face mimicry and perception, after targeted in the right M1 and the right primary somatosensory cortex with continuous theta burst stimulation (cTBS), compared to targeted in the vertex, whereas males showed no TMS effects (Korb et al., 2015). In terms of chronic treatment effects, a meta-analysis of rTMS on major depression patients revealed a dependency effect of sex, showing that females have higher response to treatment (Kedzior et al., 2014). However, it is unclear whether rTMS treatment yields a comparable gender difference in drug abuse populations.

TMS induced cortical plasticity discrepancy in male and female rarely studied, it is unclear whether male and female respond equally to TMS. Two pilot studies for cocaine dependents included two females in 18 subjects and one female in 18 subjects (Bolloni et al., 2016; Martinez et al., 2018), respectively. This defect was solved in a recent study, which reported rTMS suppresses craving in a group of female methamphetamine dependents (Liu et al., 2018). The group of female methamphetamine dependents were treated by 10 Hz rTMS stimulation in the left dorsolateral prefrontal cortex (DLPFC), for a duration of 20 times in 4 weeks with 2000 pulses single session. The results showed that the cue-induced craving score significantly decreased in the treated group, compared to the untreated group, with lasting effects until another month in follow-up. The study sufficiently demonstrated the effectiveness of craving reduction in female groups with rTMS, which is consistent with the male groups (Liu et al., 2017).

One priority for future research from the present study is to include the menstrual cycle of female dependents, which is inevitably included in 1-month course of treatment. Females have a higher sensitivity for cocaine in the follicular phase, and attenuate in the luteal phase (Bobzean et al., 2014). It may be regulated by progesterone levels, which are consistent with cortical plasticity induced by paired TMS and more inhibition in the luteal phase (high progesterone level) compared to the follicular phase (low progesterone level) (Smith et al., 1999). Individual genotypes would probably be tested before treatment in the future. For instance, the Val66Met (a type of brain-derived neurotrophic factor gene) carry an individual response unstable to TMS compared to Val66Val individuals (Cheeran et al., 2008). Another factor has been ignored is response rate to non-invasive brain stimulation (NIBS) in subjects, and the number that has been confirmed is 40% (Lopez-Alonso et al., 2014).

One limitation of the study is that it excludes the involvement of a male group. Male and female subjects have difference in reasons of using drugs, addictive behavior, and peaking of craving progress. In the initial stage, men's drug intake is more influenced by their peers, while women's drug intake is more related to their mental state (Becker and Chartoff, 2019). Female drug users alter from casual use to compulsive use more rapidly than males, and have a higher craving for drugs and relapse (Bobzean et al., 2014). Moreover, females progress more mental health symptoms during treatment. These are also important factors influencing treatment effects. Another limitation the lack of longer follow-ups (e.g., relapse, craving). The author conducted a 2 month follow-up in the present study, and found that the treatment effect was still valid (Liu et al., 2018). It is insufficient to assess the treatment effect of TMS for substance dependents, especially when the treatment effectiveness may differ in males and females.

In short, the present study demonstrated that rTMS can also reduce the craving of female methamphetamine dependents. In the future, we need to re-evaluate the similarities and differences between gender differences in treatment and brain function assessment by more clinical, cognitive and physiological indicators and protocols.

## Author Contributions

YZ drafted the manuscript. YZ and HZ revised it. WH was in charge for the final version. All authors read and approved the submitted version.

### Conflict of Interest

The authors declare that the research was conducted in the absence of any commercial or financial relationships that could be construed as a potential conflict of interest.
